# Reading Touch Screen Storybooks with Mothers Negatively Affects 7-Year-Old Readers’ Comprehension but Enriches Emotional Engagement

**DOI:** 10.3389/fpsyg.2016.01728

**Published:** 2016-11-16

**Authors:** Kirsty M. Ross, Rachel E. Pye, Jordan Randell

**Affiliations:** ^1^Department of Psychology, University of WinchesterWinchester, UK; ^2^School of Psychology and Clinical Language Sciences, University of ReadingReading, UK

**Keywords:** reading comprehension, shared reading, touch screen, scaffolding, developing readers, emotional engagement

## Abstract

Touch screen storybooks turn reading into an interactive multimedia experience, with hotspot-activated animations, sound effects, and games. Positive and negative effects of reading multimedia stories have been reported, but the underlying mechanisms which explain how children’s learning is affected remain uncertain. The present study examined the effect of storybook format (touch screen and print) on story comprehension, and considered how level of touch screen interactivity (high and low) and shared reading behaviors (cognitive and emotional scaffolding, emotional engagement) might contribute to comprehension. Seven-year-olds (*n* = 22) were observed reading one touch screen storybook and one print storybook with their mothers. Story comprehension was inferior for the touch screen storybooks compared to the print formats. Touch screen interactivity level had no significant effect on comprehension but did affect shared reading behaviors. The mother–child dyads spent less time talking about the story in the highly interactive touch screen condition, despite longer shared reading sessions because of touch screen interactions. Positive emotional engagement was greater for children and mothers in the highly interactive touch screen condition, due to additional positive emotions expressed during touch screen interactions. Negative emotional engagement was greater for children when reading and talking about the story in the highly interactive condition, and some mothers demonstrated negative emotional engagement with the touch screen activities. The less interactive touch screen storybook had little effect on shared reading behaviors, but mothers controlling behaviors were more frequent. Storybook format had no effect on the frequency of mothers’ cognitive scaffolding behaviors (comprehension questions, word help). Relationships between comprehension and shared reading behaviors were examined for each storybook, and although length of the shared reading session and controlling behaviors had significant effects on comprehension, the mechanisms driving comprehension were not fully explained by the data. The potential for touch screen storybooks to contribute to cognitive overload in 7-year-old developing readers is discussed, as is the complex relationship between cognitive and emotional scaffolding behaviors, emotional engagement, and comprehension. Sample characteristics and methodological limitations are also discussed to help inform future research.

## Introduction

Touch screen storybook apps for smartphone and tablet devices offer an interactive multimedia reading experience for children, with animations, music, sound effects, games, and oral narration accompanying the story text. Surveys by Common Sense Media (2013) in the US and the National Literacy Trust (Formby, 2014) in the UK report that just under a third of children have read books on touch screens, but this figure is likely to increase given the rapid growth in children’s access to touch screens. Latest figures from the UK show that 73% of children had access to tablets at home in 2015, up from only 14% in 2012, with 5- to 7-year-olds experiencing the largest increase in access since 2014 (Ofcom, 2015).

The present study examined the effects of touch screen storybooks and the level of touch screen interactivity on 7-year-old children’s story comprehension in shared reading contexts. The aim was to explain the comprehension effects with reference to a rich set of data and observations, including data on children’s liking of the storybooks, their format preferences, and their home reading environments, and observations of general shared reading activities, cognitive and emotional scaffolding behaviors, and positive and negative emotional engagement. Seven-year-olds have been somewhat under-represented in recently published studies of the effects of touch screens on literacy development where there has been a greater focus on toddlers’ and preschoolers’ beginning and emergent literacy (e.g., Merchant, 2015; Kirkorian et al., 2016; Neumann, 2016). However, children’s experiences with the interactive multimedia features of touch screen storybooks are particularly interesting to examine at this age because they are on the cusp of independent reading due to greater fluency and improved comprehension skills (Schwanenflugel et al., 2004), but they continue to benefit from reading with a supportive adult (Clark, 2007; Mudzielwana, 2014).

The effect of interactive multimedia features on children’s story comprehension has been subject to considerable research in recent years, but rapid changes in technology have also been taking place. Much of the existing experimental literature is based on older computer technologies which lack a touch screen interface but have other interactive and multimedia features to varying degrees, including oral narration, animations, sound effects, and hotspots (albeit activated by a mouse). We use the term e-book in this paper to refer in general terms to storybooks on electronic devices (including computers, e-readers, electronic consoles), but we specify where studies used touch screen technology. One relatively recent meta-analysis by Zucker et al. (2009) found that e-books in general have small to moderate effects on comprehension outcomes, though much of the evidence was based on experimental studies of children in the pre-reading or early stages of reading. However, this meta-analysis and other recent reviews of the literature (Miller and Warschauer, 2014; Bus et al., 2015) have concluded that the effects of e-books are neither consistently positive nor negative, and more needs to be done to pull apart the effects of different interactive features, the reading context, and participant characteristics on comprehension.

The dramatization of the story through animations and sound effects is thought to have potential to enhance children’s story comprehension by facilitating dual coding of verbal and non-verbal story information (Paivio, 1986, 2008). Paivio’s dual coding theory postulates that non-verbal stimuli might trigger questions and inferences about the verbal stimuli, resulting in deeper understanding due to interconnections between verbal and non-verbal processing. In support of this theory, studies by Verhallen and colleagues found that animations and sound effects in narrated e-books enhanced 5-year-old children’s story understanding and expressive vocabulary learning in comparison to e-books with static visuals (Verhallen et al., 2006; Verhallen and Bus, 2010). These studies were strictly controlled: the animations and sound effects were in close temporal contiguity to the story and the experimenter controlled the activation of features. When 7-year-olds were allowed to control the interactive features themselves in a study by Ricci and Beal (2002), superior comprehension of animated and narrated e-books was also found in comparison to audio books, despite the fact that some of the animations were a diversion from the main story. Another study by Smeets and Bus (2014) found that interactive animations and non-interactive animations in narrated e-books had no beneficial or detrimental effects on 4- to 5-year-olds comprehension compared to e-books with static visuals, but vocabulary learning was enhanced by interactive word definition features. Thus from these studies of children’s independent reading of e-books, it appears that interactive animations and sound effects have at least no detrimental effect on comprehension of e-books and perhaps a positive effect if well-designed.

When children’s comprehension of interactive animated e-books is compared to their comprehension of print books, the benefits of the e-book features are less evident. Two studies report that 4- to 6-year-old children’s comprehension of e-books with interactive animations, sound effects, and oral narration was comparable to their comprehension of printed books read by an adult experimenter (De Jong and Bus, 2004; Korat and Shamir, 2007). However, when interactive animations and games had low congruence with the story, children’s attention was diverted from story content toward the interactive features, resulting in less complete story retellings than for print stories read by an adult (De Jong and Bus, 2002). The interactive features of e-books could be cognitively overloading young readers when they read independently. According to cognitive load theory (Sweller, 2005), multimedia features may cause children to switch between processing the story text and processing other information. This switching may exceed their processing capacity and result in cognitive overload, with detrimental effects on learning. Young children may be particularly prone to cognitive overload due to their immature cognitive and attentional skills (Courage et al., 2015).

In everyday shared reading contexts, parents often demonstrate some degree of cognitive scaffolding to support children’s reading development and comprehension skills (Bruner, 1981; Sénéchal and LeFevre, 2002). Cognitive scaffolding behaviors, such as comprehension questioning and encouraging discussion, can also be trained, for example in the dialogic reading approach first introduced by Whitehurst et al. (1988). Various studies have demonstrated that this structured, scaffolding approach to reading with children leads to benefits in storytelling (Marjanovič-Umek et al., 2012), receptive language and attention (Vally et al., 2015), and vocabulary (Niklas and Schneider, 2015). The efficacy of these behaviors at promoting language learning from printed books declines considerably as children reach 4- to 5-years-old (as evident from the meta-analysis by Mol et al., 2008), but they may still benefit children’s general reading development and attitudes (Niklas and Schneider, 2013).

The nature and importance of parents’ cognitive scaffolding behaviors during shared reading deserves re-examination in the context of touch screen story books. The greater complexity of interactive multimedia reading, and the potential for cognitive overload, may mean that children will continue to benefit from parental support at older ages. A potential problem, however, might be parents’ perceptions of, and attitudes toward, touch screen reading. Despite parents having some concerns that animations, games, and hotspots could distract children from learning, they also seem to consider touch screen reading as being particularly suited to children’s independent reading because of the support from digital pronunciation and audio narration features (Vaala and Takeuchi, 2012).

Several recent studies have examined comprehension effects in shared reading contexts, where children read with a parent or other supportive adult. A study by Krcmar and Cingel (2014) found that the touch screen format by itself (in the absence of any interactive features, animations, or sounds) adversely affected the story comprehension of 2- to 5-year-olds compared to reading print storybooks, and resulted in fewer parent and child comments and questions about story content. The limited scaffolding by parents did not explain the lower comprehension scores, but comprehension was negatively affected by talk about the book format and environment, which happened more often in the touch screen condition. Chiong et al. (2012) also found that reading books on touch screens resulted in parents and their 3- to 6-year-old children engaging in less talk about story content and more non-content talk, but comprehension was only negatively affected by reading on an interactive touch screen and not by reading on a non-interactive touch screen. Parish-Morris et al. (2013) investigated the effects of electronic console books (where interactive sounds and games were activated by button presses) when parents read with their 3- and 5-year-olds. Parents engaged in less content-related talk and more behavior-related talk compared to reading printed stories, and 3-year-olds story recall was poorer, but the effect of age on comprehension was unclear because of ceiling effects for the 5-year-olds.

Krcmar and Cingel (2014) explained pre-school children’s poorer comprehension of touch screen storybooks in a shared reading context by drawing on Fisch’s (2000) cognitive capacity model for learning from screens. Children are thought to have limited cognitive capacity to process the narrative of the story, so non-content related talk acts to increase the cognitive load on children and reduce the resources available to process story content. Parents do not seem to have effective strategies to ameliorate the distractions of technology during shared reading, which is understandable given that sharing e-books is still a relatively infrequent activity compared to reading print books. Children’s familiarity with technology does not appear to diminish the distractions of technology in shared reading contexts; instead, children with greater experience of touch screen technology in Krcmar and Cingel’s (2014) study had poorer comprehension of touch screen storybooks, perhaps because they associated the technology with playing games rather than reading. More studies are needed to understand if school-aged children, who are becoming more skilled at following the narrative of a story, are better able to cope with the distractions of technology.

Although there is evidence that parents and children engage in distracting talk when reading on touch screens, which detracts from comprehension, some studies have reported more positive findings in relation to cognitive scaffolding behaviors. Lauricella et al. (2014) found that although parents offered help with the interactive features of an e-book at the expense of word definitions, verbal interactions were otherwise very similar across formats, and format did not affect 4-year-old children’s comprehension. Cognitive scaffolding seems to be particularly effective when delivered by teachers or other trained adults in the school environment as demonstrated in a study by Segal-Drori et al. (2010) in which 5- to 6-year-old children’s emergent word reading skills were tested after reading interactive animated e-books and print books with an adult who was trained to support word learning. Children made greatest progress in the e-book condition suggesting that interactive features can enhance children’s learning during shared reading if the adult effectively scaffolds children’s processing of the verbal information, perhaps relieving some of the processing burden.

Good quality adult support seems to be important if children are to effectively comprehend storybooks with interactive multimedia features, and quality is affected by emotional scaffolding behaviors as well as the cognitive scaffolding behaviors already discussed. Parents rate the emotional dimensions of shared reading – fostering an enjoyment of books and having a close and enjoyable time with the child – as more important than cognitive stimulation and fostering of reading development (Audet et al., 2008). Positive shared reading experiences as children are learning to read also predict better reading outcomes in later life and greater interest in reading (e.g., Baker et al., 2001; Hood et al., 2008; Hume et al., 2015), and children’s enjoyment and motivations to read are positively related to reading attainment (e.g., Baker and Wigfield, 1999; Wang and Guthrie, 2004; Taboada et al., 2009; Petscher, 2010; Clark and De Zoysa, 2011; McGeown et al., 2015; Clark, 2016). Given the long-reaching effects of positive shared reading experiences, it is important to examine how new reading technologies affect emotional scaffolding behaviors during shared reading. The potential tension between parents who prefer print books (Zickuhr, 2013; Rideout, 2014) and children who have more positive attitudes toward e-books (Vaala and Takeuchi, 2012) may adversely affect the emotional aspects of shared reading. Researchers are beginning to examine moment to moment emotional responses during adult reading (Graesser et al., 2012) and multimedia learning (Chung et al., 2015) to explore interrelationships between emotion, cognition and learning, and this is a promising area for further research into children’s experiences with new reading technologies.

Observational studies which consider children’s and parents’ positive and negative emotional engagement (as evident from emotional expressions) when they read touch screen storybooks or e-books together are hard to find, but some studies have considered general patterns of positive and negative engagement during shared reading. One small scale study found that some parents actively discouraged their 6- to- 7-year old children’s attention to interactive features (McNab and Fielding-Barnsley, 2014), and such discouragement could result in negative patterns of engagement. Other studies have found that interactive features promoted positive engagement between parents and their 4-year-olds during story reading (Lauricella et al., 2014), and between children, their peers and teachers during school literacy activities (Flewitt et al., 2015). In contrast, Chiong et al. (2012) found no difference in the positive and negative engagement of parents and their 3- to 6-year-old children when reading on touch screen and in print, although the stories were relatively short. It seems likely that reading interactive multimedia storybooks will prompt both positive and negative emotions, and more research is needed to understand the implications for children’s comprehension.

In the present study, we examined the effect of storybook format (touch screen or print) and touch screen interactivity (high or low) on 7-year-old children’s story comprehension, liking of the story, format preferences, and shared reading behaviors (including general shared reading activities, cognitive and emotional scaffolding behaviors, and positive and negative emotional engagement). The highly interactive touch screen storybook had many hotspot-activated features, including games, animations, and sound effects, in addition to sophisticated computer-generated animations and a musical soundtrack which played automatically. The less interactive touch screen storybook had hotspot activation of sentence narration and sound effects, but only static illustrations. Story comprehension was expected to be lower for touch screen formats and lowest for the highly interactive touch screen format, based on previous research about the potentially distracting nature of incidental features such as games, and because of concerns about cognitive overload and the difficulty of task switching between games and reading.

Cognitive and emotional scaffolding behaviors and emotions were observed during shared reading with the aim of further understanding the mechanisms by which storybook format and interactivity influence comprehension. Touch screen storybooks, and the highly interactive storybook in particular, were expected to negatively affect both the time that dyads spent talking about the story and mothers’ cognitive scaffolding of story comprehension by asking comprehension questions, due to the distractions from the interactive features. Despite expected negative effects on cognitive scaffolding behaviors, children were expected to like touch screen storybooks more than print storybooks and to express more positive emotion during shared reading because of their engagement with the touch screen features. The effects of format and interactivity on mothers’ emotional scaffolding behaviors and emotions were expected to be less straightforward because the interactive features might provoke both enjoyment and tensions between the dyads as the focus shifted between the story, hotspot activation and (in the highly interactive story) games; hence, no directional hypotheses were made.

Children’s general reading abilities and the home reading environment were also examined to provide further context for the interpretation of the results; for example, poorer readers might be expected to require greater supportive behaviors from parents than better readers, and differences in access to touch screens could explain differences in comprehension.

## Materials and Methods

### Participants

Participants were recruited through adverts placed in *Primary Times* magazine in the South East of England (Hampshire and Berkshire editions) in July 2015. Twenty-seven mother–child dyads participated in the study, but data from five dyads was removed due to failing to finish reading a story (*n* = 2) and incomplete video recordings (*n* = 3), leaving a final sample of 22 dyads. Child participants (14 females, 8 males) ranged in age from 6 years 4 months to 7 years 10 months (mean age = 7 years 1 month, *SD* = 6 months). Mothers ranged in age from 25 to 46 years (mean age = 37, *SD* = 6). Education levels of mothers varied: high school (*n* = 7), Bachelor’s (*n* = 12), Masters (*n* = 1) and Doctoral (*n* = 2).

### Materials and Measures

#### Reading Abilities

Children’s reading abilities were assessed with the York Assessment of Reading Comprehension: Passage Reading Primary (YARC Primary; Snowling et al., 2009); a standardized measure of reading across three dimensions - reading rate, accuracy and comprehension – and normed to a UK sample. Children first completed the Single-Word Reading Test (SWRT) as a measure of their decoding ability, and the SWRT score was used to determine their starting level on the YARC passage reading task. Children were timed as they read aloud two YARC passages with errors corrected and counted by the assessor.

#### Questionnaires

Children’s home reading environment was assessed using the Reading Environment Questionnaire (Powell and Chesson, unpublished). The first part of the questionnaire consisted of seven items to assess literacy at home, including the number of children’s print and electronic books at home and the frequency of children’s reading activities (rated on a 5-point scale: 1 = never, 2 = seldom, 3 = sometimes, 4 = often, 5 = very often). The second part of the questionnaire consisted of five items to assess home activities in general. Of relevance to this study was the number of hours in a typical day spent engaging in games and learning activities on electronic devices (rated on a 5-point scale: 0, 1, 2, 3, 4+ h) and the number of electronic devices that children had access to at home.

Children’s liking of each storybook was recorded immediately after reading on a 4-point scale (1 = not at all, 2 = not much, 3 = a little, 4 = a lot). At the end of the second visit, children were asked if they preferred to read storybooks on electronic devices or in print.

#### Storybooks

Two storybooks were selected for the study: *The Prince’s Bedtime* (TPB; Oppenheim, 2006) and *The Fantastic Flying Books of Mr. Morris Lessmore* (ML; Joyce, 2012). The storybooks were chosen because they matched our key criteria: available in both print and touch screen formats; not best-sellers and therefore unlikely to have been read by participants previously; suitable for 7-year-old children; and the touch screen formats of these two books varied in level of interactivity.

The two storybooks were comparable in the number of words (TPB = 732, ML = 729), the number of unique words (TPB = 329, ML = 294), and the mean number of letters per word (TPB = 5.04, ML = 5.24). Comparison was made to a small selection of graded Oxford Owls storybooks (levels 7–11) designed for the target age range, and the chosen storybooks were broadly comparable to this sample on word measures. The touch screen versions of the storybooks were available on the iOS platform: TPB was available through the Me Books app (2015), and ML was a standalone app published by Moonbot Studios (2012). None of the children in the study had previously read the storybooks in print or touch screen format.

The touch screen format of ML had a high level of interactivity, with hotspot activation of animations and story-relevant sound effects (1–12 hotspots per page) and five interactive games (up to 43 hotspots per game). The five games stepped out of the ML story to a degree and involved: writing in a blank book; completing a jigsaw; playing a tune on a piano; moving letters to create words and photographing the created words; and controlling the character’s flight through movement of the iPad. Hotspot activation was prompted to some extent because the page-turning icon did not appear until some hotspots had been activated, and occasionally animations drew attention to the hotspots. ML also featured computer generated animations (mostly congruent with the story) and music which played automatically on most pages without any hotspot activation. There was an option for continuous narration (not activated by hotspots) but this feature was turned off for this study because it was not suited to a shared reading context where mothers are supporting children’s developing reading skills.

The touch screen format of TPB had a low level of interactivity compared to ML, with hotspot activation of sentence narration, story-relevant sound effects, and character speech which expanded on the story (2–11 hotspots per page), but no hotspot- activated animations or games. Hotspot activation was not prompted in any way in TPB, and the digital pages could be turned even if no hotspots had been activated. All illustrations in TPB were static, and there was no background music.

#### Story Comprehension Questions

Children’s comprehension of each story was assessed with nineteen questions in chronological order written in the following styles: picture (x4), multiple choice (x3), short-answer (x3), true/false (x5) and cloze (x4; the child completes a sentence with the final missing word). These questions were piloted in a local primary school with 6- to 7-year-old children (*n* = 19) to ensure that the questions for each story were matched for difficulty. The answers to comprehension questions were scored as correct or incorrect, and correct answers were totaled to give a comprehension score for each story.

#### Design

The storybooks and related reading comprehension questions were delivered in a 2 × 2 design (story – TPB or ML; and format – print or touch screen) and counterbalanced across participants so that each child read one story in one of the formats and the other story in the other format. Thus, each child participated in one of the TPB conditions – TPB print or TPB touch screen low interactivity [TPB TS(LI)] – and one of the ML conditions – ML print or ML touch screen high interactivity [ML TS (HI)]. There were 11 children per condition.

### Procedure

Participants were visited in their home by a researcher on two occasions within a 2-week period (mean time between visits = 5 days). At the beginning of the first visit, each child’s reading rate, reading accuracy, and comprehension skills were assessed using the YARC. After the YARC, each child read a story with their mother, followed by questions about liking of the storybook and story comprehension. Mothers completed the reading environment questionnaire between visits. At the second visit, each child read a second story with their mother, followed by questions about liking of the storybook, format preference, and story comprehension.

Each child read one of the stories on an iPad (provided by the researcher) and one story in print format, in counterbalanced order. Children were asked to read the story aloud and mothers were asked to listen and to offer help where necessary, as if they were supporting their child to read a story brought home from school for the purposes of home learning. Basic guidance was given on how to turn pages in the touch screen storybooks, where required, but no other instructions were given about the interactive features.

Shared reading sessions were recorded using a Panasonic HDC-SD41 High Definition Video Camera placed on a tripod in front of the dyad. The researcher left the room while the stories were being read to avoid being a distracting presence. Observations and data collection ceased when the mother or researcher noted that the child was unduly tired or distressed by the reading activity (two participants whose data was later removed from the study).

Ethical approval for the study was granted by the Ethics Committee at the University of Winchester. Mothers gave written informed consent for their own and their child’s participation and they were fully debriefed at the end of the study. Children were also verbally informed about the study and verbally consented to take part.

#### The Observational Coding Schemes

Four coding schemes were created for the study: shared reading activity, mothers’ cognitive and emotional scaffolding behaviors, child emotion and mother emotion. A summary of the main codes and their definitions is provided in **Table 1**. Event durations were recorded for the shared reading activities and for the child and mother emotion coding schemes. In addition to the main codes, these coding schemes also had codes for *other* (activities or behaviors which did not fit into the main codes) and *obscured* (when poor visibility or audibility made it difficult to decide what was happening), though in practice these were rarely used (less than 0.5% of observed seconds). These coding schemes were mutually exclusive and exhaustive, meaning that each moment in the entire shared reading session was coded. Event frequency was recorded for mothers’ cognitive and emotional scaffolding behaviors (nominal duration of 1 s per event). This coding scheme was mutually exclusive but not exhaustive, since only events relevant to the coding scheme were coded.

**Table 1:** The observational coding scheme.

Codes	Definition
**Shared reading activity**	
Read	Mother or child read the story while the other listens; includes short pauses while turning pages or while appearing to silently read
Story talk	Mother or child talk about the story, including the meanings of words, the characters, the pictures, the plot, and thoughts and feelings about the story
Touch screen	Mother or child talk about, touch, or look at animations, hotspot activated features, or games; swiping to turn pages, without any disruption to the reading activity, is not included

**Cognitive and emotional scaffolding**	
Comprehension	Mother asks a comprehension question about the story, including the characters, the pictures, the plot
Words	Mother helps with the pronunciation or meaning of a word
Technical	Mother offers specific verbal instructions or guidance to help the child with the touchscreen activities (including turning the pages), e.g., “press this,” “if you do this, that happens”
Praise	Mother praises the child in relation to the shared reading activity, including touchscreen use, e.g., “Well done,” “Excellent,” “You worked that out!”
Control	Mother attempts to exert control over the child’s activity and uses command words, e.g., “Stop doing that,” “Move on now,” “Hurry up.” We classed control as emotional scaffolding because of the negative emotional tone, despite mothers’ positive intention to redirect children’s attention back toward the story.

**Child emotion; Mother emotion**	
Positive	Positive facial expressions of smiling, laughter, or surprise are displayed in response to the reading task
Neutral	Calm and attentive to the reading task
Negative	Negative facial expressions of boredom, frustration, confusion, anger, or anxiety are displayed in response to the reading task

The coding schemes were piloted on a small sample of the videos by the first author (KR) and the other coders before being finalized. KR coded the shared reading activities coding scheme and trained the four other coders to use the other coding schemes (SS coded child emotion, RR coded mother emotion, HG and SH coded scaffolding behaviors). Videos were viewed in Windows Media Player and coding began when the dyads started to read or talk about the story (including the front cover) or engage in the touch screen activities. Coding finished when the dyads stopped reading or talking about the story or engaging in any touch screen activities. Coders recorded code onset times and the final offset time in Microsoft Word before the data was transferred to the GSEQ software program (General Sequential Querier; Bakeman and Quera, 2011) for analysis.

Inter-observer reliability was tested by second coding the videos for 4 of the 22 participants, which accounted for 16% of the total observation time. The second author (RP) was the second coder for the shared reading activities coding scheme and KR was second coder for the other coding schemes. Two measures of reliability were calculated in GSEQ, in line with Bakeman and Quera’s (2011) recommendations: time-unit kappa with tolerance (±2 s), which measures the length of agreements and disagreements; and event alignment kappa, which measures agreement about the onset of events (tolerance 5 s, overlap 80%). GSEQ computed the value of each time-unit kappa twice, once with each observer as the first observer.

The inter-observer reliability results are summarized in **Table 2**. Reliability was good to excellent for all coding schemes: time-unit kappas ranged from 0.83 to 0.96, event alignment kappas ranged from 0.71 to 0.85.

**Table 2:** Inter-observer reliability scores for the observational coding schemes.

	Time-unit kappa, with tolerance (±2 s)	Time-unit agreement	Event alignment kappa with tolerance (5 s, 80% agreement)	Event agreement
Shared reading activity	0.94–0.95	98–98%	0.85	90%
Child emotion	0.89–0.96	99–100%	0.84	91%
Mother emotion	0.86–0.87	98–98%	0.83	90%
Mother scaffolding	0.83–0.86	99–99%	0.71	82%

*Time-unit kappa is calculated twice, once with each observer entered first.*

### Statistical Analysis

Data were checked for normality using the Shapiro–Wilk test and by inspection of the skewness and kurtosis values and histograms. The YARC and story comprehension scores approximated normal distributions; therefore, this data was analyzed with parametric statistical tests. The liking and observational data deviated from normality; therefore, this data was analyzed with non-parametric Mann–Whitney *U* tests, with comparisons made between formats for each of the two storybooks in turn. Two-tailed significance values are reported, unless otherwise stated. Spearman’s correlations were used to examine the relationships between the story comprehension data and the liking and observational data because of the non-parametric nature of some of the data. The alpha level was 0.05 for all statistical tests.

## Results

### Children’s Reading Abilities

The mean reading abilities of the child participants, as measured by the YARC, were above average: mean standard score for reading accuracy = 120.32 (*SD* = 9.04), mean standard score for reading rate = 114.23 (*SD* = 11.41), mean standard score for comprehension = 111.91 (*SD* = 7.98). The overall mean YARC score ranged from 99.67 to 127.67 (mean = 115.48, *SD* = 7.78) meaning that all children were within the average to above average range. The mean number of words read correctly in the SWRT was 37.95, *SD* = 10.03.

Correlations between YARC and SWRT scores and comprehension scores were examined by story (**Table 3**) and by format (**Table 4**). TPB comprehension was positively correlated with SWRT and most of the YARC scores (*p*s < 0.01), with the exception of YARC rate, but ML comprehension did not significantly correlate with the YARC and SWRT scores. Comprehension of print storybooks positively correlated with SWRT and most of the YARC scores (*p*s < 0.05), with the exception of YARC rate. Comprehension of touch screen storybooks positively correlated with YARC accuracy (*p* = 0.03), but not with the other YARC and SWRT scores.

**Table 3:** Intercorrelations between reading ability scores and story comprehension for ML and TBP storybooks (*n* = 22).

	2	3	4	5	6
1. ML comprehension	0.360	0.183	0.284	0.299	0.328
2. TPB comprehension		0.659^∗∗^	0.395	0.549^∗∗^	0.602^∗∗^
3. YARC accuracy			0.505^∗^	0.514^∗^	0.756^∗^
4. YARC rate				0.504^∗^	0.636^∗∗^
5. YARC comprehension					0.409
6. SWRT					

*^∗^*p* < 0.05, ^∗∗^*p* < 0.01.*

**Table 4:** Intercorrelations between reading ability scores and storybook comprehension in print and touch screen formats (*n* = 22).

	2	3	4	5	6
1. Print comprehension	0.337	0.433^∗^	0.414	0.439^∗^	0.631^∗∗^
2. Touch screen comprehension		0.462^∗^	0.234	0.329	0.316
3. YARC accuracy			0.505^∗^	0.514^∗^	0.756^∗∗^
4. YARC rate				0.504^∗^	0.636^∗∗^
5. YARC comprehension					0.409
6. SWRT					

*^∗^*p* < 0.05, ^∗∗^*p* < 0.01.*

### Reading Environment

Mothers estimated that there were 61–80 children’s print books in the house on average, with no reports of fewer than 20 print books. Availability of children’s e-books was more limited with 12 mothers reporting none and 10 reporting between 1 and 20. Mothers reported reading print books to their child significantly more often than they read books on electronic devices (*t*(21) = 14.77, *p* < 0.001); with paper books ‘often’ read (mean = 3.9), and e-books ‘never’ or ‘seldom’ read (mean = 1.27). Similarly, children independently read print books significantly more often (mean = 4.3) than e-books (mean = 1.6; *t*(21) = 8.81, *p* < 0.001).

Mothers reported that children spent less than an hour per day on computer games (mean = 0.41 h, *SD* = 0.6) and learning activities on electronic devices (mean = 0.55 h, *SD* = 0.6). There was an average of 6.9 electronic devices (e.g., laptops, PCs, smartphones) in the home, of which 2.6 on average were available for children to use.

Correlations between reading environment and story comprehension were examined. Since no predictions were made about how the reading environment would affect comprehension of print and touch screen story books, correlations were corrected using stepwise Bonferroni corrections. Only two relationships were significant: a negative relationship between the frequency of independent reading of books on electronic devices and comprehension of the touch screen storybooks (*r* = -0.546, *p* = 0.009), and a positive relationship between the frequency of reading print books independently and comprehension of printed books (*r* = 0.534, *p* = 0.01).

### Story Comprehension

The effects of story and format on story comprehension were examined with an ANOVA. There was a significant main effect of story, *F*(1,20) = 5.076, *p* = 0.036, $\eta_{p}^{2}$ = 0.202, with higher comprehension scores for ML (mean = 12.82, *SD* = 3.34) than TPB (mean = 11.96, *SD* = 2.52). There was also a significant main effect of format, *F*(1,20) = 7.31, *p* = 0.014, $\eta_{p}^{2}$ = 0.268, with comprehension scores higher for the print formats (mean = 13.27, *SD* = 1.95) than for the touch screen formats (mean = 12.82, *SD* = 1.99).

**FIGURE 1:**
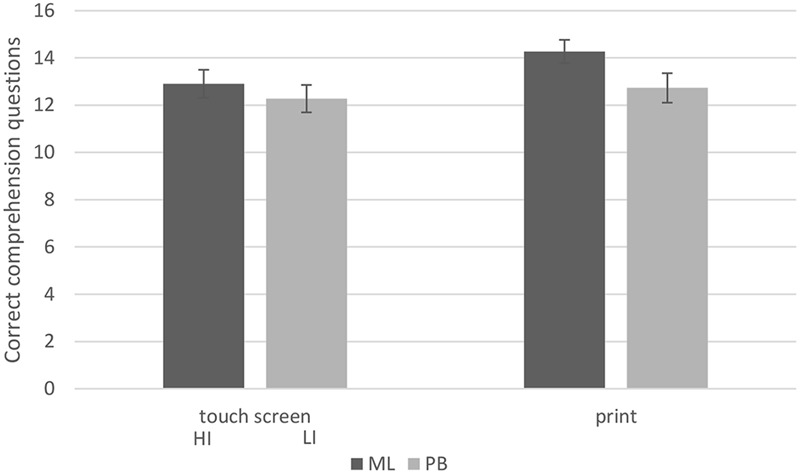
**Mean story comprehension accuracy (+SE) for ML and TPB stories, in print and touch screen format.** HI, high interactivity. LI, low interactivity.

Story comprehension was expected to be lowest in the ML TS(HI) condition, due to the high interactivity and the presence of games that were incidental to the story, but there was no significant interaction between story and format, *F*(1,20) = 0.43, *p* = 0.52, $\eta_{p}^{2}$ = 0.021. *Post hoc* tests showed no difference in comprehension scores between ML print (mean = 14.27, *SD* = 1.62) and ML TS(HI) (mean = 12.90, *SD* = 1.97), *t*(20) = 1.773, *p* = 0.092, nor between TPB print (mean = 12.73, *SD* = 2.05) and TPB TS(LI) (mean = 12.27, *SD* = 1.85), *t*(20) = 0.546, *p* = 0.591. See **Figure 1**.

### Children’s Liking of Stories and Format Preference

We hypothesized that children would like the touch screen storybooks more than the print storybooks but this hypothesis was not supported. Children’s median liking scores were 4 (“a lot”) for ML print and TPB print and 3 (“a little”) for ML TS(HI) and TPB TS(LI), but there was no significant difference in liking by format for the ML storybook (*U* = 43.00, *p* = 0.787, one-tailed) or the TPB storybook (*U* = 48.00, *p* = 0.781, one-tailed).

Correlations between reported liking and comprehension were examined by story and by format. There was a significant correlation between liking of TPB and comprehension of TPB (ρ = 0.447, *p* = 0.037), but no significant correlation between liking of ML and comprehension of ML (ρ = 0.381, *p* = 0.088). Liking scores for each format did not significantly correlate with comprehension of stories in the same format (touch screen: ρ = 0.353, *p* = 0.116; print: ρ = 0.261, *p* = 0.240).

When children were asked to state their preferred format for storybook reading after they had read both formats, the majority indicated no preference (*n* = 17), four preferred print books, and one child expressed a preference for reading on a touch screen tablet.

### Observations of the Shared Reading Experience

Children and mothers were video-recorded as they read two stories with their mothers over two sessions which resulted in 731 min of observations (mean per dyad = 33.24 min, *SD* = 13.59). Children read the majority of each story aloud while mothers helped with difficult words and phrases, and occasionally mothers took turns reading alternate pages when children were becoming tired. A team of coders analyzed the observations for the duration of different types of shared reading activities, the duration of children and mothers’ positive and negative emotions, and the frequency of mothers’ cognitive and emotional scaffolding behaviors.

#### Shared Reading Activities

The time spent engaging in three different shared reading activities – reading, talking about the story, and touch screen interaction – is summarized by format for each storybook in **Table 5**.

**Table 5:** Median durations (minutes) of shared reading activities for touch screen and print formats of two storybooks.

	ML Storybook			TPB Storybook		
	Print	TS(HI)	Significance	Print	TS(LI)	Significance
Read	8.80	8.22	ns	10.77	8.43	ns
Story talk	2.32	1.22	^∗^	2.37	1.98	ns
Touch screen activity		8.28			0.37	
Overall duration	11.67	19.73	^∗∗^	12.27	12.98	ns

*ML, The Fantastic Flying Books of Mr. Morris Lessmore. TPB, The Prince’s Bedtime. TS(HI), touch screen with high interactivity. TS(LI), touch screen with low interactivity. Significance is based on Mann–Whitney *U* tests. ns, not significant, ^∗^*p* < 0.05, ^∗∗^*p* < 0.01.*

We hypothesized that touch screen reading would negatively affect the time spent talking about the story compared to reading the print format, particularly where there was high touch screen interactivity. This hypothesis was partially supported because time spent talking about the story was lower for ML TS(HI) than ML print (*U* = 32.00, *p* = 0.033, one-tailed), but there was no difference in time spent talking about the story between TPB TS(LI) and TPB print (*U* = 52.00, *p* = 0.303, one-tailed).

The dyads spend considerably longer interacting with touch screen activities in the ML TS(HI) condition (median = 8.28 min) than in the TPB TS(LI) condition (median = 0.37 min; *U* = 5.00, *p* < 0.001). This meant that the overall length of the shared reading session was significantly longer for ML TS(HI) than ML print (*U* = 20.00, *p* = 0.008), but there was no difference between the TPB formats (*U* = 58.00, *p* = 0.870). For both storybooks, the time spent reading the story did not differ by storybook format (*U*s > 47.00, *p*s > 0.375).

#### Cognitive and Emotional Scaffolding Behaviors

The frequencies of mothers’ cognitive scaffolding behaviors (comprehension questions, word help, technical help) and emotional scaffolding behaviors (praise, control) were observed throughout the shared reading sessions. The median frequencies of scaffolding behaviors are summarized by storybook format in **Table 6**. For praise and control, median frequencies are also summarized by type of shared reading activity (either reading or story talk combined or touch screen activities). This was not relevant for the cognitive scaffolding behaviors because the nature of the coding scheme meant that nearly all of the observations of comprehension questions and word help occurred when the dyads were reading and talking about the story and all of the technical help occurred during touch screen activities. Where the medians are low (0–2), further descriptive data is provided to aid the interpretation of the significant results below. We hypothesized that the touch screen format would negatively affect the frequency of comprehension questions, but no other hypotheses were made for the effect of format on scaffolding behaviors.

**Table 6:** Median frequencies of mothers’ cognitive and emotional scaffolding behaviors during shared reading activities for touch screen and print formats of two storybooks.

	ML Storybook			TPB Storybook		
	Print	TS(HI)	Significance	Print	TS(LI)	Significance
**Cognitive scaffolding**						
Comprehension	6	3	ns	2	2	ns
Words	12	15	ns	14	15	ns
Technical		2			0	

**Emotional scaffolding**						
Praise						
Read/Story talk	0	1	^∗^	2	1	ns
Touch screen activity		0	^∗†^		0	ns^†^
Overall frequency	0	2	^∗∗^	2	1	ns
Control						
Read/Story talk	1	0	^∗^	0	0	ns
Touch screen activity		1	^∗∗†^		0	ns^†^
Overall frequency	1	2	ns	0	1	^∗^

*ML, The Fantastic Flying Books of Mr. Morris Lessmore. TPB, The Prince’s Bedtime. TS(HI), touch screen with high interactivity. TS(LI), touch screen with low interactivity. Significance is based on Mann–Whitney *U* tests. ns, not significant, ^∗^*p* < 0.05, ^∗∗^*p* < 0.01. ^†^The frequency of scaffolding behaviors during touch screen activities is compared to zero.*

#### Cognitive Scaffolding

There was no evidence to support the hypothesis that the frequency of comprehension questions was negatively affected by the touch screen format, because there were no significant differences in frequency by format for either storybook (*U*s > 53.00, *p*s > 0.143, one-tailed). The frequency of word help did not differ significantly by storybook format for either storybook (*U*s > 55.00, *p*s > 0.718). Technical help with touch screen features was observed significantly more often in the ML TS(HI) condition (median = 2; 10 of 11 mothers helped 67 times) than in the TPB TS(LI) condition (median = 0; 3 of 11 mothers helped 3 times; *U* = 13.00, *p* = 0.001).

##### Emotional scaffolding

Praise was observed significantly more frequently in the ML TS(HI) condition (median = 1; 9 of 11 mothers praised 49 times) than in the ML print condition (median = 0; 2 of 11 mothers praised 15 times; *U* = 22.50, *p* = 0.007). This difference can be explained by a higher frequency of praise when dyads were reading and talking about the story in the ML TS(HI) condition (median = 1; 8 of 11 mothers praised 35 times) compared to the ML print condition (median = 0; 2 of 11 mothers praised 14 times; *U* = 29.50, *p* = 0.025), and also by praise during touch screen activities in the ML TS(HI) condition (median = 0; 5 of 11 mothers praised 11 times; frequency was significantly greater than zero, *U* = 33.00, *p* = 0.0014). For the TPB storybook, there was no significant difference in the frequency of praise by format, either overall or when looking at different types of shared reading activities (*U*s > 47.00, *p*s > 0.363).

Control was observed relatively infrequently but was more frequent during the overall shared reading session for the TPB TS(LI) condition (median = 1; 7 of 11 mothers displayed 35 controlling behaviors) than for the TPB print condition (median = 0; two mothers displayed three controlling behaviors; *U* = 32.00, *p* = 0.034). No significant difference by format was found for times when the dyads were reading and talking about the story (*U* = 44.00, *p* = 0.188), and the frequency of control was not significantly greater than zero during TPB touch screen activities (Mann–Whitney *U* = 44.00, *p* = 0.069). For the ML storybook, there was no significant difference in the frequency of control by format for the overall shared reading session (*U* = 56.50, *p* = 0.0785), but for times when the dyads were reading and talking about the story, there were significantly fewer controlling behaviors in the ML TS(HI) condition (median = 0; 1 of 11 mothers displayed two controlling behaviors) than in the ML print condition (median = 1; 7 of 11 mothers displayed 17 controlling behaviors; *U* = 28.00, *p* = 0.013). The frequency of controlling behaviors during ML touch screen activities was significantly greater than zero (median = 1; 7 of 11 mothers displayed 13 controlling behaviors; *U* = 22.00, *p* = 0.002).

#### Child and Mother Emotion

The duration of children’s positive and negative emotions were coded for the overall shared reading session and were also examined by type of shared reading activity (reading and talking about the story combined or touch screen activities). The median durations of children’s and mothers’ positive and negative emotions and the relative duration of these emotions (as a percentage of total observation time) are summarized in **Table 7**. It was important to determine if story format affected the total duration of emotions during the shared reading sessions, but the effect of story format on length of the shared reading session also had to be considered; hence, relative durations were also analyzed. We hypothesized that the touch screen storybooks would positively affect the duration of children’s positive emotions, but we made no hypotheses about the effects on children’s negative emotions or mothers’ emotions.

**Table 7:** Median duration (seconds) and median relative duration (% of observed time) of children’s and mothers’ emotions during shared reading activities for touch screen and print formats of two storybooks.

	ML storybook			TPB storybook		
	Print	TS (HI)	Significance	Print	TS(LI)	Significance
**Child positive emotion**						
Read/Story talk	10	19	ns	43	15	ns
Touch screen activity		48	^∗∗∗†^		0	^∗†^
Overall duration	10	68	^∗∗^	43	26	ns
*Overall relative duration*	1.68%	5.76%	ns	5.83%	4.11%	*ns*

**Child negative emotion**						
Read/Story talk	0	1	^∗^	2	0	ns
Touch screen activity		0	ns^†^		0	ns^†^
Overall duration	0	1	^∗^	2	0	ns
*Overall relative duration*	0.00%	0.10%	^∗^	0.23%	0.00%	ns

**Mother positive emotion**						
Read/Story talk	34	26	ns	103	30	^∗^
Touch screen activity		123	^∗∗∗†^		0	^∗†^
Overall duration	34	189	^∗^	103	36	ns
*Overall relative duration*	5.55%	12.41%	ns	9.35%	6.00%	ns

**Mother negative emotion**						
Read/Story talk	0	0	ns	0	0	ns
Touch screen activity		0	^∗†^		0	ns^†^
Overall duration	0	2	ns	0	0	ns
*Overall relative duration*	0.00%	0.14%	ns	0.00%	0.00%	*ns*

*ML, The Fantastic Flying Books of Mr. Morris Lessmore. TPB, The Prince’s Bedtime. TS(HI), touch screen with high interactivity. TS(LI), touch screen with low interactivity. Significance is based on Mann–Whitney *U* tests. ns, not significant, ^∗^*p* < 0.05, ^∗∗^*p* < 0.01, ^∗∗∗^*p* < 0.001. ^†^The duration of emotions during touch screen activities is compared to zero.*

##### Child positive emotion

For the ML storybook shared reading sessions, children expressed positive emotions for a significantly longer total duration in the ML TS(HI) condition (median = 68 s) than in the ML print condition (median = 10 s; *U* = 22.00, *p* = 0.005, one-tailed), supporting the hypothesis that touch screen storybooks would positively affect children’s positive emotions. There was a marginally significant difference by format in the relative duration of children’s positive emotions for the ML storybook, with relative duration directionally greater in the ML TS(HI) condition (median = 5.76%) than in the ML print condition (median = 1.68%; *U* = 35.00, *p* = 0.051, one-tailed). The differences by format can be explained by the positive emotions expressed by children during the touch screen activities of the ML TS(HI) condition (median = 48 s; duration significantly greater than zero, *U* = 5.50, *p* < 0.001). When the dyads were reading and talking about the story, there was no significant difference between the ML TS(HI) and ML print conditions (*U* = 56.00, *p* = 0.767) in the duration of positive emotions.

For the TPB storybook shared reading sessions, there was no significant difference in the duration of children’s positive emotions between the TPB TS(LI) condition and the TPB print condition (*U* = 54.50, *p* = 0.651, one-tailed). Directionally, the difference was opposite to the hypothesized effect of touch screen storybooks, with the duration of positive emotions lower for the TPB TS(LI) condition (median = 26 s) than for the TPB print condition (median = 43 s). The relative duration of children’s positive emotions did not differ significantly between the TPB TS(LI) condition and the TPB print condition (*U* = 53.50, *p* = 0.674, one-tailed), though the relative duration was shorter for the TPB TS(LI) condition (median = 4.11%) than for the TPB print condition (median = 5.83%). When the dyads were reading and talking about the story, there was no significant difference by TPB storybook format in the duration of children’s positive emotions (*U* = 42.50, *p* = 0.236), but the duration of children’s positive emotions was significantly greater than zero during TPB touch screen activities (median = 0; 5 of 11 children expressed positive emotion for 250 s; *U* = 33.00, *p* = 0.014).

##### Child negative emotion

Children expressed negative emotions for relatively brief durations, and some did not express any negative emotions at all (*n* = 13). Median durations ranged from 0 to 2 s, so further descriptive data is provided below to aid interpretation of the significant results.

For the ML storybook shared reading sessions, children expressed negative emotion for significantly longer in the ML TS(HI) condition (6 of 11 children expressed 85 s of negative emotion) than in ML print condition (1 of 11 children expressed 2 s of negative emotion; *U* = 34.00, *p* = 0.035). The relative duration of children’s negative emotion was also greater in the ML TS(HI) condition (median = 0.1%) than in the ML print condition (median = 0.0%; *U* = 34.50, *p* = 0.035). The difference between conditions was driven by a significantly longer duration of negative emotion when reading and talking about the story in the ML TS(HI) condition (6 of 11 children expressed 70 s of negative emotion) than in the ML print condition (1 of 11 children expressed 2 s of negative emotion; *U* = 32.00, *p* = 0.023). The duration of children’s negative emotions during touch screen activities in the ML TS(HI) condition was not significantly greater than zero (*U* = 49.50, *p* = 0.148).

For the TPB storybook shared reading sessions, the duration of children’s negative emotions did not differ significantly between the TPB TS(LI) condition and the TPB print condition (*U* = 37.50, *p* = 0.080), though directionally the duration of negative emotions was shorter for the TPB TS(LI) condition (2 of 11 children expressed 52s of negative emotion) than the TPB print condition (6 of 11 children expressed 127 s of negative emotions). There was no significant difference in the relative duration of children’s negative emotions between the TPB TS(LI) condition and the TPB print condition (*U* = 37.50, *p* = 0.080). When considering only the times when the dyads were reading and talking about the story, there was no significant difference between the TPB conditions (*U* = 37.50, *p* = 0.080), nor was the duration of children’s negative emotions during TPB touch screen activities significantly greater than zero (*U* = 60.50, *p* = 1.00).

##### Mother positive emotion

During the ML storybook shared reading sessions, mothers expressed positive emotions for significantly longer durations in the ML TS(HI) condition (median = 189 s) than in the ML print condition (median = 34 s; *U* = 29.00, *p* = 0.039). When the relative duration of mothers’ positive emotions was examined, it was directionally greater for the ML TS(HI) condition (median = 6% of observation time) compared to the print condition (median = 2%), but the difference was not significant (*U* = 34.50, *p* = 0.086). The difference in duration between the two ML conditions can be explained by the positive emotions expressed by mothers during the touch screen activities of the ML TS(HI) condition (median = 123 s; duration significantly greater than zero; U = 0, p < 0.001). When the dyads were reading and talking about the story, there was no significant difference between the two ML conditions in the duration of mothers’ positive emotions (*U* = 55.00, *p* = 0.718).

During the TPB storybook shared reading sessions, the duration of mothers’ positive emotions did not differ significantly by condition (*U* = 38.50, *p* = 0.148), nor did relative duration (*U* = 53.50, *p* = 0.643). When dyads were reading and talking about the story, mothers expressed positive emotions for significantly shorter durations in the TPB TS(LI) condition (median = 30 s) than in the TPB print condition (median = 103 s; *U* = 28.50, *p* = 0.036). The duration of mothers’ positive emotions during TPB touch screen activities was significantly greater than zero seconds (median = 0; 4 of 11 mothers displayed positive emotions for 264 s; *U* = 38.50, *p* = 0.032).

##### Mother negative emotion

Mothers expressed negative emotions for relatively brief durations, and some did not express any negative emotions at all (*n* = 14). Median durations ranged from 0 to 2 s, so further descriptive data is provided below to aid interpretation of the significant results.

For the ML storybook, there were no significant differences in the duration of mothers’ negative emotion between the ML TS(HI) and ML print conditions either, overall or when the dyads were reading and talking about the story, nor was there any difference in relative duration (*U*s > 48.00, *p*s > 0.223). Mothers expressed negative emotions during the ML touch screen activities for a duration that was significantly greater than zero (five mothers for a total of 38 s; *U* = 33.00, *p* = 0.014).

For the TPB storybook, there were no significant differences between the TPB TS(LI) and TPB print conditions, either in duration or relative duration of mothers’ negative emotions, nor in the duration of mothers’ negative emotions when the dyads were reading and talking about the story (*U*s > 54.00, *p*s > 0.606). Mothers did not express negative emotions during the TPB touch screen activities for a duration significantly longer than zero (*U* = 49.50, *p* = 0.148).

#### Comprehension and Shared Reading Observations

Correlations between children’s comprehension and shared reading behaviors were examined for each storybook separately, regardless of format, to further understand the different patterns observed for the two storybooks. Comprehension of the ML story (highly interactive when in touch screen format) was significantly negatively correlated with the length of the shared reading session (ρ = -0.448, *p* = 0.036). There was a marginal negative correlation between time taken to read the ML story (excluding time spent talking about the story and engaging with touch screen activities) and comprehension (ρ = -0.385, *p* = 0.076), but no other correlations were significant (ρs < -0.358, *p*s > 0.102). Comprehension of the TPB story (less interactive when in touch screen format) was significantly negatively correlated with the frequency of controlling behaviors of mothers (ρ = -0.483, *p* = 0.023). There was a marginal negative correlation between the frequency of praises and TPB comprehension (ρ = -0.403, *p* = 0.063), but no other correlations were significant (ρs < 0.320, *p*s > 0.146).

Children’s reading abilities may have affected some of the shared reading behaviors, so we examined correlations with the mean YARC scores. There were significant negative correlations between reading ability and three variables: the frequency of word help (ρ = -0.0611, *p* = 0.003), the frequency of mothers’ controlling behaviors (ρ = -0.499, *p* = 0.018), and the time taken to read the story (ρ = -0.693, *p* < 0.001).

## Discussion

The present study examined the effects of reading touch screen storybooks with different levels of interactivity on 7-year-old readers’ comprehension, storybook liking, format preferences and shared reading behaviors. Detailed observations of shared reading behaviors and activities were collected from 22 mother-child dyads as they read two storybooks together, one in print and one in touch screen format, with the aim of further understanding the underlying mechanisms behind any effects on comprehension. As a group, our 7-year-old participants were above-average readers, with good access to electronic devices in the home, good access to print books in the home, but limited or no access to books on electronic devices in the home.

Children’s comprehension was inferior for the touch screen formats of two storybooks (when the results were pooled), which was in line with the hypothesized effect of reading format on comprehension, but there was no evidence that the level of touch screen interactivity had an effect. The level of touch screen interactivity did affect shared reading behaviors, such that the expected reduction in time spent talking about the story and the expected increase in children’s positive emotional engagement was only evident for the highly interactive touch screen storybook. The highly interactive touch screen storybook had other notable effects including significantly longer shared reading sessions (due to several minutes of engagement with touch screen activities) and increased negative emotional engagement from children (alongside increased positive emotional engagement). The hypotheses that touch screen reading would negatively affect the frequency of mothers’ comprehension questions and positively affect children’s liking of the storybooks were not supported.

The effects of touch screen reading on mothers’ emotional scaffolding behaviors and emotional engagement were expected to be complex because of tensions between ‘fun’ interactive features and mothers’ learning orientation during shared reading. The highly interactive touch screen storybook increased mothers’ positive emotional engagement and the frequency of praise, including praise during reading and talking about the story, while the less interactive touch screen storybook increased the frequency of controlling behaviors, compared to print storybooks. Touch screen storybooks did not significantly affect mothers’ negative emotional engagement during the overall shared reading session, but some mothers did express negative emotions for brief though notable durations during the highly interactive touch screen activities.

### Comprehension

Shared reading of touch screen storybooks resulted in inferior story comprehension compared to reading the same stories in print format, as predicted. This finding supports previous research that interactive multimedia features can interfere with children’s comprehension in shared reading contexts (Chiong et al., 2012; Parish-Morris et al., 2013). However, the expected interaction between touch screen interactivity level and comprehension was not found, and when comprehension was examined for each storybook individually, neither the highly interactive nor the less interactive touch screen storybook affected children’s comprehension in comparison to the print storybook. The small sample size unfortunately limited the ability of the study to detect small effects on comprehension for the individual storybooks, so there was no support for the hypothesis that highly interactive touch screen features (including games with limited story congruence) are more detrimental to comprehension than less interactive and more congruent touch screen features.

Our comprehension findings lend some support to the theory that the presence of any interactive multimedia features places greater demands on information processing compared to reading in print, and risks cognitive overload due to the need to switch between different types of tasks (Sweller, 2005; Bus et al., 2015; Courage et al., 2015). The findings offer no support to Paivio’s (2008) dual-coding approach which suggests that interactive features congruent with the story would aid comprehension. The interactive sound effects, character speech and sentence narration of the less interactive touch screen storybook in our study were congruent with the story, but no positive effect on comprehension was found.

Three findings are particularly relevant to understand how processing the story plot might be affected by touch screen features: (1) the highly interactive touch screen storybook resulted in significantly longer shared reading sessions because of the touch screen interaction time; (2) the length of the shared reading session was negatively related to comprehension across both storybooks, and (3) touch screens did not affect time taken to read the story itself. These findings lead us to conclude that children’s ability to process the plot as a coherent whole was being disrupted by touch screen activities interspersed between reading the story, which in turn meant that it took longer to reach the end of the story despite reading time being unaffected. It would be interesting to examine if prompting parents and children to recap the story after significant periods of touch screen interaction would ameliorate the negative effects on comprehension.

The children in our study were 7-year-old developing readers with above average reading abilities but that did not appear to protect them from the detrimental effects on comprehension which may have resulted from cognitive overload and task switching. Children in this age range have immature cognitive and attentional skills (see Courage et al., 2015, for a review) but to develop as readers they are required to constantly and accurately map multi-modal information – of a known sound (phoneme) to an unfamiliar visual code (letter or grapheme)- and to build an understanding of the text. It is also worth noting that the children in our study read aloud the majority of the each story, while mothers helped with word pronunciation, and meaning, but only read aloud occasionally. This would have significantly increased children’s cognitive load compared to listening to the story being read by their mother or a narrator, as is typical in studies with younger children. Given these challenges, it is unsurprising that developing readers in shared reading contexts, however proficient in comparison to peers, are susceptible to cognitive overload and task-switching effects.

Children’s home reading environment had some influence on comprehension of the storybooks in our study. As would be expected, there was a strong relationship between the frequency of reading printed books independently and the comprehension of the two storybooks in printed format, but perhaps surprisingly there was a negative relationship between the frequency of reading e-books independently and comprehension of the two storybooks in touch screen format. This effect of technology experience is similar to that reported by Krcmar and Cingel (2014), and is worthy of further exploration with a larger sample. Perhaps young children with more experience of reading interactive books pay greater attention to interactive features and games and less attention to the story. Older children (8–16 years old) have been found to make greater progress in their reading skills when they more frequently accessed e-books at school (Picton and Clark, 2015), but e-books designed for older school children do not typically feature the cutting edge multimedia animations and games that are appearing in the latest touch screen storybook apps for emergent and developing readers. As reading on interactive touch screens becomes more common, it will be important to understand how longer term exposure affects the early development of reading comprehension skills.

### Cognitive Scaffolding

Shared reading of interactive touch screen storybooks did not affect mothers’ cognitive scaffolding of comprehension in comparison to print storybooks, contrary to our expectation. The expected negative effect on the length of time that the dyads talked about the story was evident, though only for the highly interactive storybook. Neither cognitive scaffolding behaviors nor duration of story talk had a significant relationship with children’s comprehension, despite comprehension being poorer for touch screen storybooks at an overall level.

Our cognitive scaffolding findings are similar to Chiong et al. (2012) who found both a combination of reduced talk about the story and poorer comprehension when reading touch screens. However, in contrast to Parish-Morris et al. (2013) and Krcmar and Cingel (2014) we found no significant reduction in comprehension questioning, nor did we find a significant reduction on word scaffolding as reported by Lauricella et al. (2014). Our child participants were at least 2–3 years older than the children in these previous studies, and mothers may have been less inclined to ask comprehension questions because of the more developed comprehension skills of 7-year-olds, particularly given the above average reading abilities of our sample.

Mothers’ most frequent cognitive scaffolding behavior in our study was support with word pronunciation and meaning, and the frequency of this behavior was negatively related to children’s reading abilities. Directive support with technical features of the touch screens was very infrequent, which could have been due to limited knowledge of touch screen features or limited interest in encouraging the use of interactive features. A motivation to support reading fluency by helping with word pronunciation and meaning may have come to the fore in all conditions of our study, because children were being observed as they read aloud. Further observations of shared reading when utilizing the oral narration function of touch screen storybooks would help to explain these observations.

While it is positive that reading on touch screens did not appear to disrupt mothers’ normal scaffolding of comprehension and word pronunciation and meaning, there appears to be untapped potential for touch screen storybooks to support and enhance these behaviors. Several studies have found that mothers’ cognitive scaffolding behaviors spontaneously occur at relatively low levels but can be increased by receiving training in dialogic reading skills (such as open-ended questions and plot expansions), with corresponding benefits for children’s literacy and enjoyment of reading (Lonigan et al., 1999; Mol et al., 2009; LaCour et al., 2013; Beschorner and Hutchison, 2016). Touch screen story books which prompt and guide the supportive behaviors of parents during shared reading could positively impact on children’s comprehension, though the effects may be more pronounced for poorer readers.

### Emotional Scaffolding

Two emotional scaffolding behaviors of mothers were examined in this study. Praise had a positive emotional tone, while control had a negative emotional tone even though mothers’ were attempting to scaffold attention to the story. These emotional scaffolding behaviors were differentially affected by the level of interactivity of touch screen storybooks. Praise was more frequent for the highly interactive touch screen storybook compared to print, while controlling behaviors were more frequent for the less interactive touch screen storybook compared to print.

Praise occurred more frequently during shared reading of the highly interactive touch screen storybook for two reasons: mothers were praising more than they did in the print condition when the dyads were reading and talking about the story, and they were also praising at a notable level during engagement with touch screen activities. The higher frequency of praise from mothers could be related to the relative ease with which children worked out the touch screen features of a novel and highly interactive storybook, including swiping to turn pages, hotspot activation and games, particularly as mothers typically only provided two instances of directive technical support. More frequent praise when reading and talking about the story might have been an attempt to positively encourage attention to the story in response to the distractions of technology, but we can only speculate because we did not ask mothers about their intentions. No relationship was found between praise and children’s comprehension. We know of no other study which specifically examined the effect of touch screen technology on praise, but our finding is consistent with Lauricella et al. (2014) who observed greater positive engagement between parents and children when reading interactive e-books.

Controlling behaviors were more frequent during shared reading of the less interactive touch screen storybook compared to print, and controlling behaviors were also negatively related to comprehension and negatively related to reading ability. Our anecdotal observations indicated that the controlling behaviors during shared reading of the less interactive touch screen storybook were often happening when children were repeatedly playing with the swipe feature to turn the page which was a distraction from the story. There was very limited engagement with the hotspots, which often went unnoticed in the static illustrations. We suspect that mothers may have been particularly motivated to direct the attention of children who needed more help with reading away from story-irrelevant touch screen features and back toward the story, but further research is needed with a wider range of reading abilities because all of the children in our sample were average or above average readers.

It is interesting that the highly interactive touch screen storybook had no significant effect on controlling behaviors even though previous research has highlighted parental concerns about interactive features with perceived low educational value, such as games (Vaala and Takeuchi, 2012). Despite these potential concerns, there was no indication that parents were using controlling behaviors to direct attention away from the highly interactive features, at least not on the first exposure to the storybook.

Emotional scaffolding of touch screen reading deserves further research attention. Praise and control were observed at relatively low levels in our study, and it may be worth looking at a wider range of parental behaviors during shared reading which promote reading enjoyment and alleviate frustrations.

### Child and Mother Emotion

Children and mothers’ demonstrated greater positive emotional engagement with the highly interactive touch screen storybook than with the same story in print, due to additional positive emotions expressed during the touch screen activities. There was no difference in children and mothers’ positive emotional engagement when they were reading and talking about the story, despite the highly interactive storybook having unprompted animations playing during these times. Thus, it appeared to be interactivity rather than the animations alone which prompted positive emotions. There was no significant relationship between positive emotional engagement and comprehension, which was somewhat surprising given that reported enjoyment of reading improves reading attainment outcomes (Clark and De Zoysa, 2011), but few studies have considered the relationship between observed emotions during reading and comprehension.

Children also demonstrated greater negative emotional engagement with the highly interactive touch screen storybook compared to the print storybook, particularly when they dyads were reading and talking about the story. When children were engaging in the touch screen activities, they did not express negative emotions for a notable duration. Mothers, in contrast, demonstrated some negative emotional engagement during the touch screen activities of the highly interactive storybook, though their negative emotional engagement was not affected by format at an overall level. Some caution must be applied to the interpretation of the negative emotional engagement findings because negative emotions were only expressed briefly by some children and mothers. From our anecdotal observations, we suggest that the pattern of negative emotional engagement observed for the highly interactive touch screen storybook appeared to be indicative of some tension created by switching between reading and talking about the story and engaging in the touch screen activities. Some children got frustrated during the story by a desire to skip ahead to the touch screen activities, and some mothers got frustrated when touch screen activities were too prolonged.

Future studies with larger samples, greater contextual analysis, and analysis of child and mother characteristics are needed to explore the underlying reasons for negative emotional engagement with touch screen storybooks and its effects. Experiencing negative emotions during reading is not necessarily detrimental because it could promote productive thinking and discussion (Graesser et al., 2012), thus potentially enriching engagement and enhancing learning where there is appropriate scaffolding, though there was no evidence of a relationship between negative emotions and comprehension in our study.

The less interactive storybook had no effect on children’s positive and negative emotional engagement, or on mother’s negative emotional engagement, perhaps because the interactive features were often missed. Mothers’ positive emotional engagement was lower for the less interactive storybook than for the print format during times when the dyads were reading and talking about the story. This finding coincides with increased control by mothers when the dyads were reading and talking about the less interactive storybook, seemingly in an attempt to redirect children’s attention from playing with the page-turning swipes. This focus on control could have reduced the opportunity for positive emotional engagement.

### Storybook Liking and Format Preferences

Children’s reported liking of the storybooks was not affected by storybook format, in contrast to our expectation and despite the observed differences in emotional engagement for the highly interactive touch screen storybook. The majority of children reported no preference for storybook format at the end of the study, which supports Jones and Brown’s (2011) finding that children showed no difference in expressed preference for or liking of e-books over print books, even after repeated exposure. Reported preferences and liking may not, however, be an entirely accurate indication of behaviors because Jones and Brown (2011) also observed that children chose to read e-books more often than print books when given the choice.

Sample characteristics may have limited the variance in the liking ratings in our study: the children had above average reading abilities overall, and children who are reading at a higher level than expected for their age are more likely than poorer readers to enjoy reading in general (Clark, 2016). We did not ask mothers for their liking and preferences, which might have helped understand their observed negative emotions and controlling behaviors.

### Limitations

The child and mother dyads were less confident with the use of touch screen devices than we had anticipated, and the dyads missed several of the interactive features. Hotspots were hardly noticed in the less interactive storybook unless they happened to be pressed accidentally, and although the on-screen prompts facilitated much more interaction with the highly interactive storybook, the hotspots and games were still not used to their full potential. The child participants had relatively little experience of reading books on electronic devices, despite using electronic devices for games and other learning activities. As a result, our study can draw conclusions about the effects of the relatively novel experience of shared reading of touch screen storybooks, but it does not demonstrate the effects of touch screen storybooks when interactive features are used to their full potential by experienced touch screen readers. It would be beneficial for future studies to provide parent–child dyads with greater exposure and training in the use of interactive features.

The analysis of the comprehension findings was complicated to some extent by the fact that children found one story (ML) easier to understand than the other (TPB), despite careful piloting of the comprehension questions to match them for difficulty. This could be resolved by creating materials specifically for the purposes of the study, where the story remains constant but the interactive multimedia features are varied. Nevertheless, there is value in examining shared reading in a naturalistic situation with real touch screen storybook apps, particularly as the sophistication of the content and features of stories such as *The Fantastic Flying Books of Mr. Morris Lessmore* is far beyond what could realistically be produced for academic research.

Sampling issues also restricted the ability of our study to explain the comprehension differences. There were only 22 mother-child dyads and 44 observations in total, and the characteristics of the sample were relatively narrow (good readers, well-educated mothers), which may have limited the variance needed to find small effects. Sample size does generally have to be sacrificed to some extent when conducting detailed observational analysis, but the richness of the data helps to provide direction to future research.

We did not consider the effects of direct on-screen touch on comprehension, since our observations were limited to time spent looking at and engaging with touch screen activities rather than counting the number of touches. Touch may further complicate the relationship between interactive multimedia storybooks and comprehension, and research is only beginning to explore how direct on-screen touch might influence children’s learning (e.g., Walsh and Simpson, 2013; Vatavu et al., 2015; Kirkorian et al., 2016).

## Conclusion

This study provides some indication that children’s inferior comprehension of touch screen storybooks in a shared reading context could be related to the increased length of the shared reading session and reduced story talk, at least when there is high interactivity, and it could also be related to more maternal control, particularly when the interactive features are less engaging. The results were complicated by the fact that format had no effect on comprehension when considering the two storybooks individually. High touch screen interactivity enriched emotional engagement and increased maternal praise, and touch screens had no detrimental effects on comprehension questioning and word help, but there was no evidence that these observations were related to comprehension. Further research with larger samples is needed to fully examine the mechanisms driving the comprehension effects.

Interactive touch screen storybooks clearly have potential to increase positive emotional engagement during shared reading, and well-designed educational features may also help to reduce the potential negative effects of task-switching and to support refocusing of attention back to the story. Our findings highlight the potential for storybook apps to include design features which directly support the comprehension and word decoding skills of developing readers, either by prompting relevant cognitive scaffolding behaviors from parents or by acting as a substitute for skilled parental support. Further studies should examine the shared reading behaviors of children with a broader range of reading abilities, since the good readers in this study did not require a great deal of cognitive scaffolding from mothers. The effects of increased exposure and confidence with touch screen technology should also be studied, since the technology is still relatively novel in the context of storybook reading.

## Author Contributions

The three authors (KR, RP, JR) jointly conceived and designed the study, and each author contributed to the drafting, revision and approval of the final paper. All authors agree to be accountable for all aspects of the work. KR led the design and piloting of the observational coding scheme, analysis of the coding data, and writing of the final paper. RP led the design and piloting of the comprehension questions, participant recruitment, and analysis of the comprehension data.

## Conflict of Interest Statement

The authors declare that the research was conducted in the absence of any commercial or financial relationships that could be construed as a potential conflict of interest.
